# Personalized Diabetes Management with Digital Twins: A Patient-Centric Knowledge Graph Approach

**DOI:** 10.3390/jpm14040359

**Published:** 2024-03-28

**Authors:** Fatemeh Sarani Rad, Rasha Hendawi, Xinyi Yang, Juan Li

**Affiliations:** Computer Science Department, North Dakota State University, Fargo, ND 58105, USA; fatemeh.saranirad@ndsu.edu (F.S.R.); rasha.hendawi@ndsu.edu (R.H.); xinyi.yang@ndsu.edu (X.Y.)

**Keywords:** digital twins, personal health knowledge graph, data integration, ontology, diabetes management

## Abstract

Diabetes management requires constant monitoring and individualized adjustments. This study proposes a novel approach that leverages digital twins and personal health knowledge graphs (PHKGs) to revolutionize diabetes care. Our key contribution lies in developing a real-time, patient-centric digital twin framework built on PHKGs. This framework integrates data from diverse sources, adhering to HL7 standards and enabling seamless information access and exchange while ensuring high levels of accuracy in data representation and health insights. PHKGs offer a flexible and efficient format that supports various applications. As new knowledge about the patient becomes available, the PHKG can be easily extended to incorporate it, enhancing the precision and accuracy of the care provided. This dynamic approach fosters continuous improvement and facilitates the development of new applications. As a proof of concept, we have demonstrated the versatility of our digital twins by applying it to different use cases in diabetes management. These include predicting glucose levels, optimizing insulin dosage, providing personalized lifestyle recommendations, and visualizing health data. By enabling real-time, patient-specific care, this research paves the way for more precise and personalized healthcare interventions, potentially improving long-term diabetes management outcomes.

## 1. Introduction

Diabetes is a complex chronic condition affecting millions globally. Successful management demands the constant monitoring of blood glucose, diet, exercise, medication, and various other factors [1]. The complexity of diabetes stems from its interplay of genetic, environmental, and lifestyle dietary influences. Effective interventions also require comprehensive knowledge about the patient, including genetics, demographics, health history, family history, lifestyle patterns, dietary habits, and real-time health data, as well as an understanding of the patient’s own knowledge and awareness of their condition. However, existing approaches often struggle with personalization, preventing complications, ensuring patient awareness, and addressing adherence issues [2]. A need exists for a comprehensive, patient-centered diabetes management system that adapts to individual needs, goals, and preferences, providing timely and accurate guidance.

Traditional research on diabetes management tends to adopt a piecemeal approach, focusing on isolated factors such as genomics, symptoms, or lab data alone. This fragmented view limits the effectiveness of interventions and hinders the development of truly personalized solutions [3].

Digital twins represent an emerging technology with the potential to transform healthcare. A digital twin is a virtual replica of a physical entity capable of modeling and simulating its behavior and environmental interactions [4]. Applied successfully in industries like aerospace and manufacturing [5], digital twins now open new possibilities for personalized medicine and precision healthcare [4].

This paper explores digital twins specifically for diabetes management, focusing on the aspects of condition prediction and control. Our approach aims to be more holistic. We propose a digital twin framework, facilitating the integration of diverse factors and empowering healthcare providers and patients to manage this complex disease comprehensively. Moreover, the accuracy of digital twins in predicting glucose levels is a testament to their potential, offering a reliable tool for managing both chronic and acute diabetes cases. Their adaptability ensures tailored applications for both Type I and Type II diabetes, demonstrating efficiency in various aspects of diabetes management. Our framework is highly efficient in managing diabetes as it provides personalized care plans based on comprehensive data, leading to improved treatment outcomes and patient self-management.

Our key contributions include the following:

- Holistic Approach: Our framework promotes a holistic diabetes management approach by integrating diverse physiological, lifestyle, social, environmental, and dietary factors.
- A patient-centric framework: We propose a framework built around personal health knowledge graphs (PHKGs) to capture the complex and evolving relationships among diverse data sources such as patient history, lifestyle, preferences, goals, and environmental factors and patients’ self-acquired knowledge.
- Data Integration and Interoperability: Our framework incorporates HL7 standards [6] to promote seamless interaction across devices, applications, programs, and institutional boundaries.
- Extensibility and Adaptability: PHKGs offer a flexible structure, allowing them to expand as new knowledge about the patient becomes available.
- Demonstrated Use Cases: We showcase the usage of the digital twin framework for real-world diabetes management applications like predicting glucose levels, optimizing insulin dosage, offering lifestyle recommendations, tailored dietary advice, and health data visualization.

The rest of the paper is organized as follows: Section 2 provides a literature review on diabetes management and the existing applications of digital twin technology in healthcare. Section 3 describes the design and implementation of our proposed framework and explains how it can create and assess digital twins for personalized diabetes care. Section 4 presents the use case study of our framework. Section 5 concludes the paper and indicates future works.

## 2. Related Works

### 2.1. Diabetes Management

While the multifaceted nature of diabetes is widely acknowledged, existing management approaches tend to adopt a fragmented view, focusing on isolated aspects of the disease. Numerous studies concentrate on identifying the genetic markers associated with an increased risk of diabetes [7]. This research helps understand disease predisposition and may open doors to future personalized therapies. The powerful impact of lifestyle factors on diabetes risk and management is well documented [8]. Interventions targeting diet, physical activity, and stress reduction are often essential components of diabetes care plans. Studies are increasingly examining the role of environmental factors as potential contributors to diabetes [9]. Factors like air pollution, exposure to specific chemicals, and access to healthy foods are under scrutiny as these may significantly influence the risk of developing diabetes and the effectiveness of treatment plans.

Diabetes research encompasses several key areas, each playing a crucial role in advancing our understanding and treatment of this complex disease. Risk prediction models seek to identify individuals at heightened risk based on genetic, lifestyle, and environmental factors, opening doors to proactive or preventative interventions [10,11]. Personalized treatment planning aims to tailor medication, diet, and exercise regimens to an individual’s unique biology and preferences, maximizing therapeutic outcomes and minimizing side effects [12,13]. Preventive strategies focus on identifying modifiable factors like diet, physical activity, and exposure to environmental toxins, aiming to reduce the likelihood of developing diabetes or to slow disease progression in those already diagnosed [14,15]. While valuable independently, the most significant impact will likely come when these research areas are united within a holistic framework, allowing for truly personalized and dynamic risk assessments, treatment plans, and preventive measures.

### 2.2. Digital Twins in Healthcare

The concept of digital twins, virtual models that mirror the real-time status and functioning of physical entities, is revolutionizing the healthcare sector. Within this context, a digital twin aims to model a patient, organ, disease process, or even a whole healthcare system, providing a precise and dynamic representation that can be used for various simulations and predictions.

Digital twins offer unprecedented opportunities for personalized treatment. These virtual replicas facilitate highly individualized care plans by integrating patient-specific data (genomic, medical history, lifestyle, and real-time sensor data). Studies like Thamotharan et al. [16] showcase how digital twins can optimize medication regimens (e.g., insulin administration), while others explore their use in surgery planning [17] and tailored device development [18,19]. Beyond individual care, digital twins can inform larger-scale public health interventions. Researchers are investigating how they might be able to model disease spread patterns, predict outbreaks [20], and simulate the impact of public health policies in specific populations. Digital twins can accelerate drug discovery and streamline clinical trials [21]. They offer the ability to test drug efficacy and safety profiles in a virtual environment, reducing time and risk while potentially tailoring therapies to specific populations [22]. Digital twins serve as powerful tools for education and training. They allow medical professionals to practice complex procedures, visualize anatomical structures, and experiment with different treatment scenarios in a risk-free, simulated environment [23,24].

While promising, digital twin technology in healthcare is still developing. Seamless data flow across various systems and devices is crucial for robust digital twin creation. Efforts continue to standardize data exchange and ensure interoperability [25,26]. As with any patient data usage, privacy, security, and the potential for biases require careful attention within digital twin frameworks [27,28].

## 3. Constructing Patient-Centered Digital Twins for Diabetes Management

**Figure 1:**
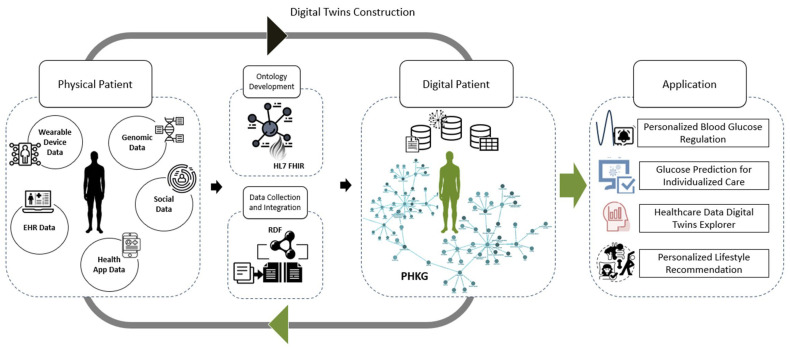
The digital twin framework.

The core objective of this research is the development of digital twins for personalized diabetes management. This involves generating dynamic virtual representations of a patient’s health state that can simulate behavior, predict outcomes, and enable personalized insights. These virtual representations are designed to be adaptive, continuously updating with real-time data to reflect the patient’s current health status accurately. Figure 1 shows the framework of the proposed digital twins. This framework integrates various data sources to construct a comprehensive model of the individual’s health. The framework comprises multiple layers, starting with ontology development, data collection and integration, personal health knowledge graph creation, and application. Details of the framework are illustrated as follows:

### 3.1. Ontology Development

The cornerstone of our approach is the development of a robust and standardized ontology meticulously aligned with HL7 FHIR standards [29] to ensure interoperability and adherence to established industry practices. This health ontology is intricately designed to offer a comprehensive vocabulary and articulate the complex relationships inherent within the personal health domain, acting as the structural foundation upon which our digital twin models are constructed.

Our methodology for ontology development embraces a systematic, top-down approach, initiating with broad health-related categories such as “Medical Condition” and progressively dissecting these into more specific subcategories like “Diabetes”. This hierarchical structure allows for a nuanced categorization of health conditions. Each concept within the ontology is enriched with properties that detail its attributes and the relationships it shares with other concepts. These are divided into object properties, which connect different concepts within the ontology, and data properties, which link concepts to specific values, facilitating a detailed and relational representation of health data.

Given the dynamic nature of medical knowledge, our ontology is crafted with flexibility in mind, enabling it to evolve in response to new medical discoveries and shifts in healthcare practices. The process of ontology evolution is triggered by the emergence of new findings, initiating a multi-step maintenance protocol that begins with the identification of necessary updates. These updates may range from the introduction of novel concepts to alterations in existing relationships within the ontology. A critical reassessment of the updated ontology’s compatibility with existing data ensures seamless integration with the pre-established data framework, maintaining the coherence and functionality of the digital twin models.

In the realm of health ontology, our approach is meticulous and fluid, allowing for the construction of a system that is not only flexible but also at the forefront of technological advancements. Establishing such a foundational framework is crucial; it serves as the backbone for the precision and dependability of our digital twin models. These models are integral to our operations, providing a reliable basis upon which we can build and improve.

Moreover, this robust framework is instrumental in elevating the level of personalization and efficacy within healthcare delivery. By laying down a solid foundation, we are able to tailor healthcare solutions to individual needs, thereby optimizing the overall healthcare experience. This commitment to enhancing personalization directly translates into more effective healthcare outcomes, ensuring that each patient receives care that is specifically designed to meet their unique health requirements.

### 3.2. Data Collection and Integration

Our approach to data collection and integration is expansive and meticulous. We tap into a wide array of healthcare data sources, including but not limited to electronic health records (EHRs), inputs from wearable devices, mobile health applications, and direct patient-generated data. Each of these sources plays a pivotal role in painting a comprehensive picture of a patient’s health landscape, offering unique insights that are integral to the construction of a detailed and accurate digital twin.

A rigorous quality control process is initiated to ensure the integrity and usability of the collected data. This critical phase addresses common data quality issues, such as missing values, inconsistencies, and outliers, which are inherent challenges in dealing with diverse healthcare datasets. Following the rectification of these issues, the data undergo a transformation process. This crucial step involves mapping the raw data onto the predefined concepts and relationships within our health ontology. The aim here is to achieve a unified and standardized representation of the data, ensuring that it aligns seamlessly with the structured framework of our ontology, thereby facilitating an accurate and effective data integration.

The cornerstone of our data integration strategy is the innovative use of the GLAV (Global–Local as View) framework [30]. This advanced framework stands out from traditional data integration approaches, such as GAV (Global as View) and LAV (Local as View), by offering a more dynamic and flexible mapping capability. The essence of GLAV lies in its ability to support bidirectional mappings, which is particularly advantageous when dealing with the voluminous and intricate nature of healthcare datasets. This flexibility is crucial for accommodating the complex interrelations and the heterogeneity inherent in healthcare data, thereby ensuring a more cohesive and comprehensive integration process.

To further refine the data integration process and enhance the accuracy of mappings, we employ Conditional Random Fields (CRFs) [31]. These advanced probabilistic graphical models are renowned for their proficiency in pattern recognition and their ability to learn complex patterns within data. By leveraging CRFs, we are able to discern and accurately map the intricate features of source data—such as column names and data types—onto the relevant concepts within our ontology. This level of precision in mapping is pivotal for ensuring that the integrated data are not only accurate but also meaningful within the context of the digital twins, enabling a richer and more nuanced representation of the patient’s health status. Through this comprehensive and nuanced approach to data collection and integration, we ensure the assembling of a rich and coherent dataset. This dataset forms the backbone of our digital twins, providing the depth and breadth of information necessary for simulating realistic and detailed virtual representations of patients’ health conditions, thereby paving the way for personalized and precise healthcare interventions.

### 3.3. Personal Health Knowledge Graph (PHKG) Construction

The construction of the personal health knowledge graph (PHKG) is a critical phase that follows the meticulous integration and transformation of health data. This pivotal transformation marks the transition of raw health data into a structured format that is amenable to semantic querying and reasoning, laying the groundwork for the robust instantiation of the knowledge graph. The PHKG is sculpted based on the intricacies of the predefined ontology, serving as a dynamic representation of a patient’s health landscape.

The instantiation process within the PHKG begins with the systematic identification and creation of specific instances for each ontological concept derived from the integrated health data. For instance, an individual blood glucose measurement recorded in the health data is instantiated within the graph as a particular manifestation of the “Blood Glucose Level” concept. This step transforms abstract ontological concepts into concrete instances that reflect real-world data points related to the patient’s health status. Simultaneously, the relationships among these instances, as delineated by the ontology through a network of object and data properties, are materialized as edges within the graph. These edges serve as the connective tissue between concept instances, weaving a complex web of relationships that mirrors the intricate, multifaceted nature of health data. For example, a “has Symptom” relationship might be instantiated to connect a “Diabetes” concept instance with a “Frequent Urination” symptom instance, thereby encapsulating the symptomatology associated with the condition within the patient’s health profile.

The PHKG transcends its role as a mere data repository, emerging as a sophisticated and integrated knowledge representation framework capable of encapsulating a wide spectrum of health-related information. This includes, but is not limited to, diagnostic information, medication regimes, laboratory results, sensor-derived data, lifestyle parameters, and subjective patient experiences. The comprehensive nature of the PHKG makes it an invaluable resource for underpinning simulations and analyses within the digital twin framework, enabling a nuanced and holistic understanding of the patient’s health dynamics.

### 3.4. Digital Twin Generation

The generation of a digital twin for each patient is a sophisticated process that harnesses the depth and breadth of the data encapsulated within the personal health knowledge graph (PHKG). This rich repository of semantically structured health data forms the bedrock upon which the digital twin is constructed, enabling a dynamic and personalized virtual representation of each patient’s health status.

The digital twin employs advanced simulation models that are meticulously calibrated using comprehensive data derived from the PHKG. These models can simulate various physiological and metabolic processes relevant to the patient’s condition, providing a virtual environment in which the consequences of different interventions can be explored. The simulation models are designed to mimic the patient’s response to various treatments, lifestyle modifications, and potential disease-progression pathways. This allows healthcare providers to visualize the potential outcomes of different therapeutic strategies, facilitating informed decision making and personalized care planning. Moreover, the models can simulate the long-term implications of these interventions, aiding in the prevention and management of potential complications.

In parallel, the digital twin leverages machine learning algorithms that are trained on the heterogeneous and comprehensive dataset provided by the PHKG. These algorithms are adept at uncovering complex patterns within the data, including subtle correlations between various health indicators, treatment responses, and environmental or lifestyle factors. By analyzing these patterns, the algorithms can generate predictive insights into the patient’s future health trajectory, identify risk factors for disease progression, and suggest preemptive measures to mitigate these risks.

The digital twin’s machine learning component is not static; it continuously evolves as new data are incorporated into the PHKG, ensuring that the twin remains up to date with the patient’s current health status and the latest medical knowledge. This dynamic learning process enhances the precision of the digital twins’ predictions and recommendations, making them increasingly personalized and accurate over time.

## 4. Application of Digital Twins

Digital twins, with their rich data integration and simulation power, unlock diverse applications for diabetes management. Here, we explore key use cases, emphasizing how digital twins’ components drive their functionality.

### 4.1. Personalized Blood Glucose Regulation [32]

**Figure 2:**
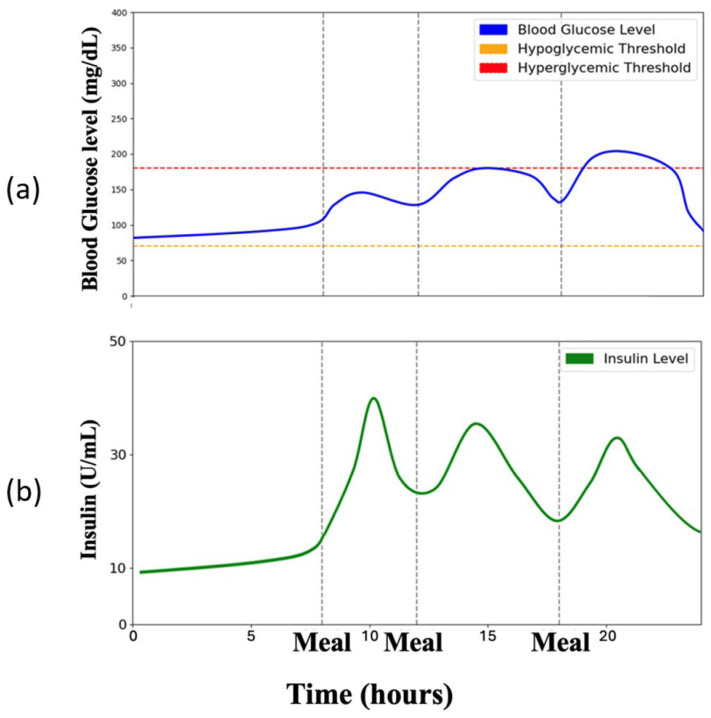
Optimized insulin dosage and resulting blood glucose control. (**a**) Simulated blood glucose trajectory under personalized insulin regimen. (**b**) Insulin doses are determined by the digital twins and administered after each meal.

- Digital Twin’s Role: The digital twin provides historical patient data and a simulation environment to train and test insulin optimization strategies.
- Algorithms: Reinforcement learning (RL), specifically the Soft Actor–Critic (SAC) algorithm, refines insulin dosages with its entropy-driven reward function. The SAC algorithm balances precision with safe exploration to find optimal solutions for the individual.
- Outcomes: The digital twin enables personalized, data-driven insulin optimization, enhancing blood glucose control while reducing risks like hyperglycemia and hypoglycemia. Our method’s efficacy was assessed based on three parameters: blood glucose concentration, the likelihood of experiencing hypoglycemia or hyperglycemia, and the duration within the euglycemic zone (70–180 mg/dL), which is considered the ideal blood glucose interval to reduce diabetes-related complications. The outcomes indicated that our approach effectively maintained blood glucose within the desired range, reducing the risk of extreme fluctuations and increasing the time spent in the euglycemic zone. This demonstrates the power of digital twins to drive individualized care. Figure 2 demonstrates the application of Soft Actor–Critic (SAC)-based reinforcement learning (RL) on a patient’s digital twin to enhance glucose level regulation, aiming to minimize the percentage of time that blood glucose level is in the risk range and stabilize overall glucose levels.

### 4.2. Glucose Prediction for Individualized Care [33]

**Figure 3:**
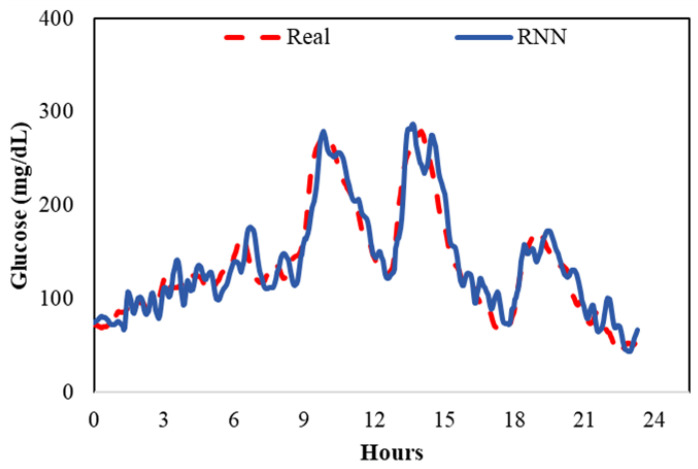
Blood glucose prediction based on the digital twin.

- Digital Twin’s Role: The digital twin framework provides a structured dataset that integrates critical patient-specific factors such as glucose trends, food intake, insulin usage, and more. This comprehensive dataset is pivotal for the development and training of effective predictive models.
- Algorithms: Recurrent Neural Networks (RNNs) are ideal for analyzing time-series data within the digital twins. These networks identify complex patterns in individual glucose trajectories.
- Outcomes: The predictive models powered by the digital twins generate individualized glucose forecasts, enabling proactive care adjustments. These personalized predictions assist both patients and healthcare providers in making informed decisions to maintain blood glucose levels within the optimal range. As depicted in Figure 3, we integrated RNNs with our digital twins to predict glucose values, achieving an average Root Mean Square Error (RMSE) of 19.83 mg/dL, which signifies the models’ precision based on the digital twins’ data. This indicates a high level of accuracy in glucose prediction, which is critical for effective diabetes management. This metric reflects the model’s ability to provide reliable forecasts that can be used in clinical settings.

**Figure 4:**
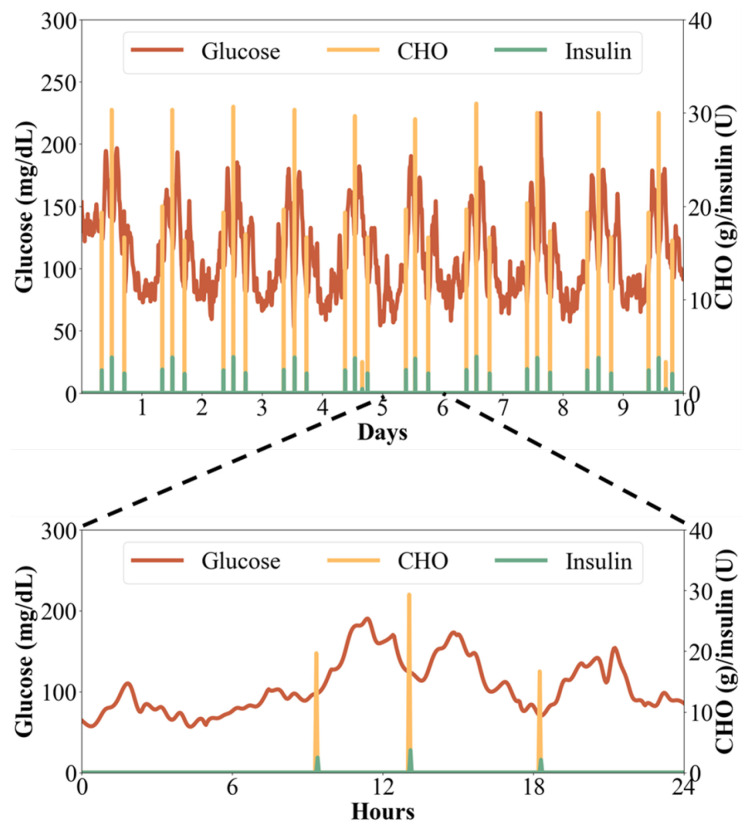
Glucose, insulin, and carbohydrate (CHO) monitoring based on the digital twin.

Figure 4 displays information gathered over a span of 10 days for an adult patient, showing their glucose, insulin, and carbohydrate (CHO) levels at 3 min intervals. Glucose levels are indicated on the left axis, while insulin and CHO levels are on the right. CHO values remain at zero except during mealtimes, and insulin levels remain stable until after meals when additional insulin is administered to manage glucose levels. The figure showcases the patient’s use of self-designed food intake and time inputs, enabling the digital twin to monitor glucose levels and adjust interventions accordingly. The digital twin offers continuous monitoring and personalized interventions based on real-time data. Moreover, it allows for detailed examination of daily or hourly data, as demonstrated by the data from day 5 to day 6 presented within a 24 h timeframe.

### 4.3. Healthcare Data Digital Twin Explorer [34]

**Figure 5:**
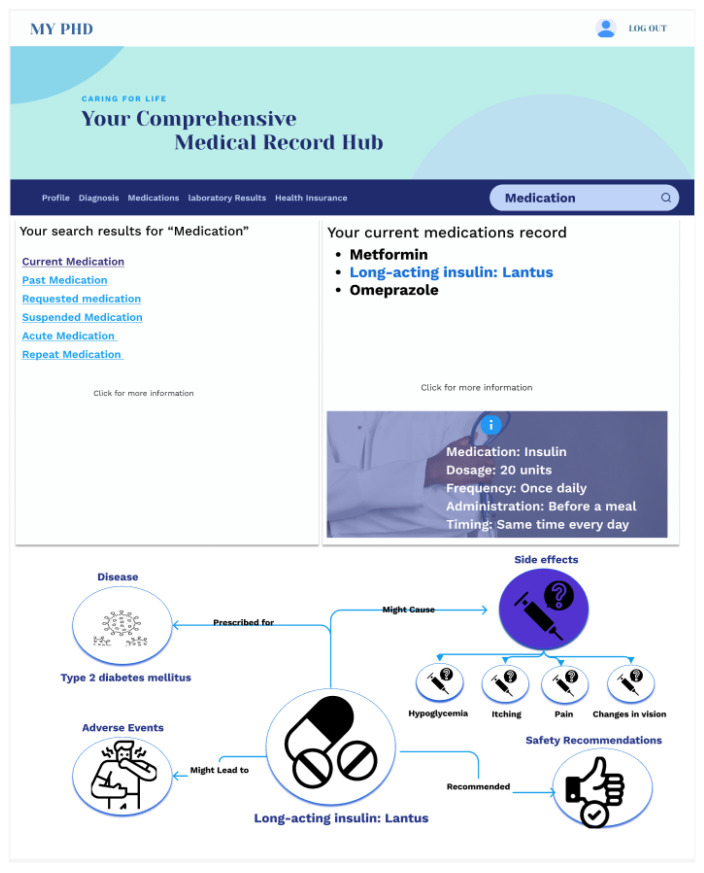
Health data explorer based on the digital twin.

**Figure 6:**
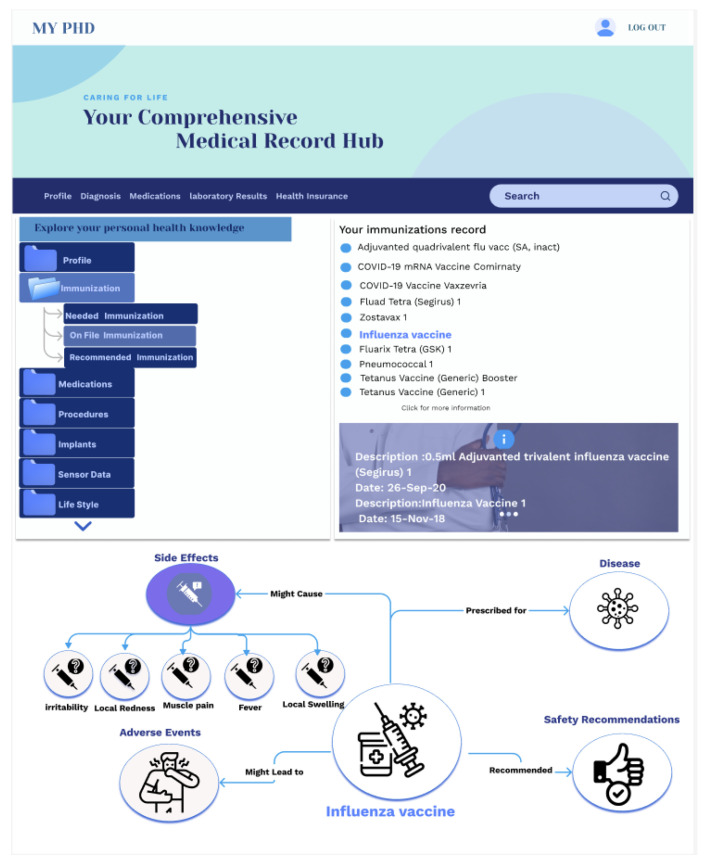
Navigation interface of explorer based on the digital twin.

- Digital Twin’s Role: The structured PHKG and its rich relationships between health concepts form the core of this application.
- Interaction Modes: As shown in Figure 5 and Figure 6, two interfaces provide flexibility:
  - Keyword Search: Natural language processing converts user queries into SPARQL for knowledge graph retrieval. Semantic links offer further exploration avenues.
  - Navigation Interface: Dropdown trees and graph visualizations allow users to explore the PHKG’s knowledge hierarchy.
- Outcomes: The digital twin serves as a powerful tool for patients, granting them the ability to comprehend their health data independently and at their own pace. This empowerment in data literacy is instrumental in fostering patient engagement and active participation in collaborative care processes. Moreover, this highlights the digital twin’s potential to enhance patient-centered care by providing a user-friendly platform for health data exploration.

### 4.4. Personalized Meal Recommendation [35]

**Figure 7:**
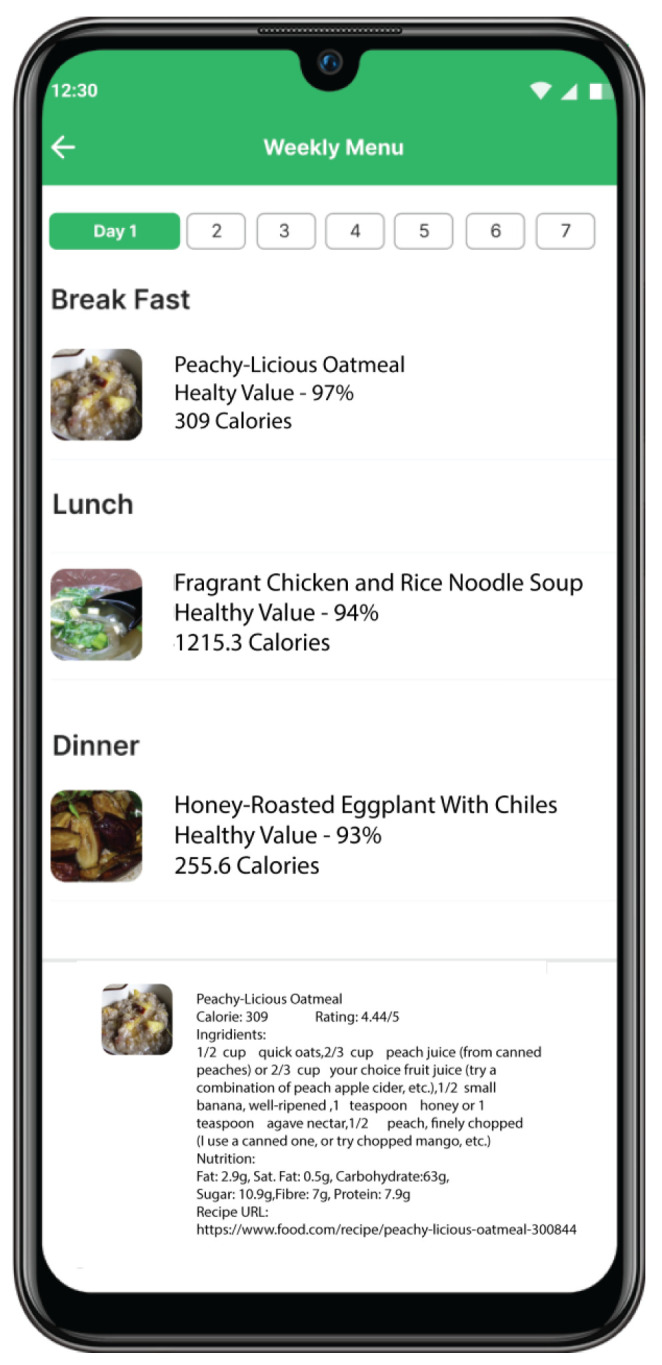
Personalized meal recommendations based on the digital twin.

- Digital Twin’s Role: The PHKG integrates information about a patient’s health condition, diabetes management plan, dietary preferences, and allergies. This comprehensive data profile fuels the meal recommendation engine.
- Logic Rules and Reasoning: Embedded within the knowledge graph are rules that reason about the patient’s health data and generate personalized meal suggestions. For example, rules might suggest meals that meet specific calorie goals, avoid allergens, and align with diabetes management guidelines.
- Outcomes: Our digital twin extends its capabilities beyond mere data provision; it delivers practical, personalized dietary recommendations that cater to each individual’s unique needs. This enables patients to make well-informed food selections that help regulate their blood sugar levels and enhance their overall health. As demonstrated in Figure 7, we have developed a mobile application that leverages our digital twin to offer individualized meal suggestions for patients with diabetes.

## 5. Conclusions

In this paper, we presented a novel approach to personalized diabetes management using digital twins and patient-centric knowledge graphs (PHKGs). We emphasized the importance of including diverse healthcare data sources while ensuring interoperability through standardized ontologies. We outlined a comprehensive methodology for constructing digital twins for diabetes management, encompassing ontology development, data collection and integration, PHKG construction, and digital twin generation. We highlighted the strengths of our approach, including patient-centricity, knowledge sharing through standardized ontologies, and the adaptability of the PHKG to incorporate new data and knowledge.

We showcased the use of the digital twins in several applications critical for diabetes management: personalized blood glucose regulation, glucose prediction for individualized care, exploration of healthcare data through the PHKG user interface, and generation of personalized meal recommendations. Our experiments indicate that the machine learning algorithms employed within the digital twin framework are highly precise in predicting and controlling glucose levels. This precision is a result of the comprehensive data and sophisticated machine-learning techniques utilized.

We plan to explore further advanced machine learning algorithms, particularly those suited for causal reasoning within the PHKG. This will enhance the digital twin’s ability to not only predict outcomes but also identify the underlying factors influencing those outcomes. Large-scale clinical trials will be crucial to validate the effectiveness of the digital twin approach in improving clinical outcomes for patients with diabetes. We envision the expansion of the digital twin framework beyond diabetes management to encompass other chronic conditions. The core functionalities and the patient-centric design principles can be adapted to other use cases within the broader healthcare domain. By continuously developing and refining the digital twin approach, we hold the potential to revolutionize personalized diabetes management, empowering patients to take an active role in their health and well-being.

## Data Availability

The datasets analyzed during this study are available from the corresponding author upon reasonable request.
